# Endothelial Progenitor Cells and NADPH Oxidase Enzyme Activity in the Development of an Aortic Aneurysm

**DOI:** 10.21470/1678-9741-2020-0458

**Published:** 2022

**Authors:** Bilge Bingol, Deniz Elcik, Sinan Kutuk, Sevil Özsoy, Saban Kelesoglu, Aydin Tuncay, Zeki Cetinkaya, Joma Sulaiman, Mehmet Tugrul Inanc, Nihat Kalay, Mustafa Yavuz Koker

**Affiliations:** 1 Department of Cardiology, Faculty of Medicine, Erciyes University, Kayseri, Turkey.; 2 Department of Immunology, Faculty of Medicine, Erciyes University, Kayseri, Turkey.; 3 Department of Cardiovascular Surgery, Faculty of Medicine, Erciyes University, Kayseri, Turkey.

**Keywords:** Thoracic Aortic Aneurysms, Dihydrorhodamine 123, Endothelial Progenitor Cells, Flow Cytometry, Disease Progression

## Abstract

**Introduction:**

Endothelial progenitor cells (EPCs) and nicotinamide adenine dinucleotide phosphate (NADPH) oxidase enzyme activity may affect the vessel wall and have a role in development of aortic aneurysms. EPCs originate from hematopoietic stem cells and can be enumerated from peripheral blood samples by flow cytometry. In this study, we aimed to evaluate the relation of EPC number and NADPH oxidase enzyme activity in the development of thoracic aortic aneurysm (TAA).

**Methods:**

Patients with TAA (n=30) and healthy individuals without TAA (control, n=10) were included in our study. Characterization and enumeration of EPC from peripheral blood samples were performed by flow cytometry with panels including markers of EPCs (CD34/CD133/CD309/CD146/CD144). Additionally, NADPH oxidase enzyme activity (capacity) was also measured by the dihydrorhodamine 123 (DHR 123) test.

**Results:**

The enumeration of EPC with CD34+/CD146+ marker showed that the number of mean EPC/106 cells was increased in the patient group (41.5/106 cells), but not in the control group (20.50/105 cells) (*P*<0.01). Additionally, patients with TAA presented significantly lower NADPH oxidase activity by DHR assay than healthy controls (mean stimulation index: 60.40± 7.86 and 75.10±5.21, respectively) (*P*<0.01).

**Conclusion:**

Our results showed that the number of EPCs is significantly higher in aortic aneurysm patients and may have a role in disease progression. The crosstalk between NADPH oxidase enzyme capacity and EPC number may be useful as a parameter to explain the clinical progression of TAA.

**Table:** 

Abbreviations, acronyms & symbols			
AAA	= Abdominal aortic aneurysm	EPCs	= Endothelial progenitor cells
ACE	= Angiotensin-converting enzyme	H_2_O_2_	= Hydrogen peroxide
ARB	= Angiotensin receptor blocker	HbA1C	= Hemoglobin A1C
BNP	= B-type natriuretic peptide	NADPH	= Nicotinamide adenine dinucleotide phosphate
BUN	= Blood urea nitrogen	NOX	= NADPH oxidase isoform
CAD	= Coronary artery disease	PMA	= Phorbol 12-myristate 13-acetate
COPD	= Chronic obstructive pulmonary disease	ROS	= Reactive oxygen products
CRP	= C-reactive protein	SI	= Stimulation index
DHR	= Dihydrorhodamine	TAA	= Thoracic aortic aneurysm
DMSO	= Dimethyl sulfoxide		

## INTRODUCTION

Aortic aneurysm is a disease that occurs as a result of the weakening of the aortic vascular wall and dilation of the aortic vessel up to 50%, usually with no symptoms, resulting in rupture of the aortic artery and death of the patient^[1]^.

Thoracic aortic aneurysm (TAA) is one of the major causes of morbidity and mortality worldwide. The etiology of TAAs is probably multifactorial, and smoking, chronic obstructive pulmonary disease, hypertension, atherosclerosis, male gender, age, body mass index, and family history are classic risk factors for this disease. In addition, genetic factors play an important role in the development of the disease^[1]^. TAAs are clinically classified into three groups as syndromic (Marfan, Loeys-Dietz, and Ehlers-Danlos), familial (non-syndromic), and sporadic^[2]^.

Another risk factor is thought to be oxidative stress and its induced endothelial dysfunction. Oxidative stress also is known to play a role in the development of aneurysm^[3]^. It is thought that the damage may happen through two main mechanisms. The two main sources of vascular reactive oxygen products (ROS) are the nicotinamide adenine dinucleotide phosphate (NADPH) oxidase found in the mitochondrial respiratory chain and smooth muscle cells. Superoxide, produced by NADPH oxidase enzyme, has a very short half-life, is converted to hydrogen peroxide (H_2_O_2_), then to hypochlorous acid by the enzyme myeloperoxidase, and takes part in apoptosis^[4]^. This apoptosis mediates endothelial damage. ROS production capacity of peripheral neutrophils can easily be measured by dihydrorhodamine 123 (DHR 123) assay, which is practically used for the diagnosis of chronic granulomatous disease^[5]^. NADPH oxidase enzyme has seven isoforms in mammals — in this case, NADPH oxidase isoform (NOX) 2, expressed in endothelial cells, plays a role in the regulation of various functions of the endothelial cell and angiogenesis. It has been shown that the change in the ratio of NOX1/NOX2 plays a role in the development of endothelial dysfunction, hypertension, and inflammation^[6]^. Stimulated NADPH activity increases the amount of intracellular superoxide radical (O-.) and H_2_O_2_ in smooth muscle cells in the vessel. As a result, the ROS amount in the vessel increases, so this stimulates various intracellular signal transduction pathways and triggers the development of TAA^[7]^.

Endothelial progenitor cells (EPCs) have proliferation, differentiation, and tissue regeneration capacity and are precursors of endothelial cells. In the event of endothelial damage or ischemia, secreted chemokine mediators induce EPCs proliferation from the bone marrow and migration to the inflammation site and joins the structure of newly formed vessels^[8]^. The aortic aneurysm may result from the disruption between the damage to the vessel wall and the repair mechanisms. EPCs are the main reservoir cells of the endothelium, playing an important role in ensuring endothelial integrity. Therefore, it can be thought that EPCs have a place in the pathogenesis of aortic aneurysms with impaired endothelial structure. There are few findings of the development of TAA disease. Although there is strong evidence that both NADPH oxidase activity and EPC balance play a role in the development of this disease, molecular mechanisms have not yet been elucidated^[7]^.

In the current report, we present a single-center study with 30 patients with TAA for the enumeration of EPCs and results of DHR assay. We compared patients and control samples for the number of EPCs and NADPH oxidase activity since these parameters have relation with the clinical progression of TAA patients.

## METHODS

### Patients and Controls

The study consisted of 30 patients who were admitted to our clinic between 2014 and 2018 and were diagnosed with TAA at the cardiology department. The local ethics committee approval and patient informative consent were obtained (2018/209, decision date: 04.04.2018). One hundred and fifty patients were involved, but only 30 patients with aortic aneurysm who met the criteria were included in this study, and 10 persons without aortic aneurysm were selected for volunteer control group. Blood samples of the patients and the control group were taken for EPC and NADPH oxidase enzyme activity analyses. In this study, the tissue samples were not taken because the patients were followed up. A total of 150 patients were evaluated with inpatient screening; 65 of these patients were excluded because of poor image quality and because there was a difference between the measurements made by two cardiologists. Collagen tissue disease, malignancy, cirrhosis, hemodialysis, cardiac-S development, pulmonary hypertension, and congenital cardiac defect were detected in 35 patients. The remaining patients were excluded because they refused to participate in the study. A control group that was completely healthy and whose echocardiographic parameters were completely normal was included. Malignancy, cirrhosis, hemodialysis, cardiac-S development, pulmonary hypertension, and congenital cardiac defect were the exclusion criteria. Electrocardiography and blood pressure measurements were performed on each patient.

### Transthoracic Echocardiography

Echocardiography was also performed on each patient to evaluate cardiac function and to measure the aortic diameter. Aorta, ascending aorta, left atrium, left ventricle, and partially right ventricle were evaluated in the parasternal long-axis window. The aortic root was measured at the widest segment of the middle sinus of Valsalva. Internal diameter measurements were taken from the widest part of the ascending aorta. Measurements in echocardiography were taken in diastole. The aortic valve and its structure (valve diseases that may be related to the ascending aorta), left ventricular diameter, and mitral valve and its structure were evaluated. Aortic valve structure, aneurysm, and ascending aortic aneurysm were used to compress other anatomic structures in the apical five-space window. The aortic valve was evaluated with color Doppler for insufficiency and stenosis.

Images were obtained from all patients and control groups using the VIVID S6 echocardiography device by monitoring at the left lateral position. Echocardiography result was reported using routine echocardiographic windows and techniques, and the distance of the widest part of the ascending aorta to the sinus of Valsalva was noted.

### Measurement of Endothelial Progenitor Cells

EPC expression was tested with CD34, CD45, CD146, CD144, and CD309 specific marker antibodies by Navios EX flow cytometry (Beckman Coulter, Brea, California, United States of America). Total leukocytes were isolated from 100 to 200 µL of blood from patients and controls using lysis of the erythrocytes in the pellet fraction with a non-fixing lysis solution, as described by Köker et al.^[5]^. 5 µL CD34 ECD, 5 µL CD146 PC5, 5 µL CD144 PE, and 5 µL CD309 PC7 were added to 12×75 mm flow tubes. 100 alkanes were added to each of the added markers and left to incubate in the dark for 20 minutes by vortexing. At the end of the period, 500 µL lysing solution was added to each tube, and the tubes were vortexed and left to incubate in the dark for 10 minutes. At the end of the period, they were centrifuged at 1500 rpm for five minutes at room temperature. The supernatant was removed. Then, 2 mL of cell washing solution was added to each tube and the previous centrifugation was repeated. After isolation, specific markers were added to flow tubes, vortexed, and incubated, then centrifuged, and all the processes performed according to the previous practice^[9^-^11]^. The analysis was completed by the enumeration of EPC in 1×10^6^ cells by special gate on the flow cytometer.

### Neutrophil Functional Assay

Total leukocytes from 100 to 200 µL of blood from patients and controls were isolated by lysis of erythrocytes in the pellet fraction with an unstable lysis solution, as described by Köker et al.^[5]^. The capacity of neutrophils to produce reactive oxygen species has been tested with the DHR experiment^[12]^. In this test, the isolated neutrophils were incubated with DHR 123 (DHR 123 preparation: 10 mg of DHR 123 and 1 mL of dimethyl sulfoxide [DMSO] were mixed, the resulting 29 mM of DHR 123 working solution was used), stimulated with phorbol 12-myristate 13-acetate (PMA) (PMA preparation: 1 mg of PMA diluted in 500 µL of DMSO and 2 µg of PMA/mL solution was obtained; 5 µL of stock solution was diluted with 2 mL of Hank’s Balanced Salt Solution and diluted with a final concentration of PMA working solution; 5 ng/mL was obtained), and analyzed by flow cytometry with Kaluza software (Beckman Coulter). The results are shown as the stimulation index (SI)^[5]^, which gives the fluorescent intensity ratio from stimulated and unstimulated neutrophils.

### Statistics

IBM Corp. Released 2012, IBM SPSS Statistics for Windows, version 21.0, Armonk, NY: IBM Corp. was used for statistical analysis. Suitability of the data for normal distribution was evaluated by histogram and Q-Q graphs and the Shapiro-Wilk test. Continuous variables between the two groups were compared using the independent *t*-test. Pearson χ^2^ analysis and Fisher’s exact χ^2^ test were used for comparison of categorical data. All tests were two‐tailed, and *P*<0.05 was considered statistically significant. The relationship between quantitative data was evaluated by Spearman’s correlation analysis.

## RESULTS

TAA patients (n=30, mean age 58.6±9.4 years), consisting of six women (mean age 58.5±12.9 years) and 24 men (mean age 58.6±8.7 years), and healthy controls (n=10, between 31-72 years), consisting of two women (mean age 58.5±19 years) and eight men (mean age 48.6±12.7 years), were included in this study. Patient and control groups were similar in terms of age and gender distribution.

While the tricuspid aortic valve was found in 26 (86.7%) of 30 patients, the bicuspid valve was seen in two (6.7%), and replaced aortic valve for aortic valve disease was also seen in two (6.7%). All the control patients had a tricuspid aortic valve. The demographic and clinical characteristics of the patient and control groups are shown in Table 1.

**Table 1:** Risk factor, echocardiogram, and laboratory findings of patient and control groups.

	Aneurysm (+) group n=30	Control group n=10	*P*-value
Age (years)	58.6±9.4	51.7±12.9	0.07
Male	81.8%	80%	0.68
Aortic diameter (mm)	48 (44-51.5)	30.9 (30-32)	< 0.01
Ejection function	56.2 (53.8-60.5)	63.3 (55-68)	0.02
Systolic blood pressure	134.3 (100-193)	122.2 (95-170)	0.06
Diastolic blood pressure	83.1±14	68.2±9.9	< 0.01
Heart rate	73.6±11.7	68±5.6	0.52
Risk factors			
Hypertension, n (%)	24 (80)	3 (30)	< 0.01
Diabetes mellitus, n (%)	12 (40)	0	0.01
Hyperlipidemia, n (%)	12 (40)	2 (20)	0.44
Smoking n (%)	12 (10)	4 (40)	0.08
COPD, n (%)	9 (30)	1 (10)	0.40
CAD, n (%)	10 (33.3)	3 (30)	0.58
Medical treatment			
Calcium antagonistic, n (%)	10 (33)	1 (10)	0.23
ACE-inhibitor, n (%)	7 (23)	1 (10)	0.65
ARB, n (%)	12 (40)	0 (0)	0.019
β-blocker, n (%)	21 (70)	2(20)	0.009
Statin, n (%)	6 (20)	2 (20)	0.65
Antiaggregant, n (%)	15 (50)	3 (30)	0.46
Anticoagulant, n (%)	3 (10)	0 (0)	0.56
CRP	6.9±11.1	1.3±1.4	< 0,01
White blood cell	7.1±1.4	6.4±1.5	0.18
Neutrophil	4.2±1.2	3.7±0.9	0.29
Lymphocyte	2.2±0.7	1.9±0.6	0.39
Monocytes	0.6±0.14	0.4±0.1	0.012
Eosinophils	0.17±0.1	0.16±0.09	0.75
Basophils	0.04±0.01	0.035±0.01	0.36
Hemoglobin	14.5±2.2	14.7±1.1	0.86
Platelets	265±70	228.5±41.4	0.08
Hematocrit	44.3±4.7	43.9±3.3	0.46
Glucose	105±14.9	95.6±13.8	0.87
HbA1C	5.8±0.59	5.4±0.32	0.02
BUN	18.2±4.7	14.7±4.7	0.03
Creatinine	0.98±0.2	0.93±0.17	0.57
Sodium	137.9±25.9	143.3±2	0.34
Potassium	4.4±0.36	4.3±0.2	0.53
Chlorine	102.6±3.1	105.1±1.9	0.05
Calcium	9.5±.32	9.5±0.33	0.05
Magnesium	0.86±0.06	0.88±0.04	0.34
Triglyceride	168.4±101.3	149.3±81.4	0.81
Total cholesterol	181.9±33.6	197.7±29.7	0.12
High-density cholesterol	41.1±10.9	43.1±7.6	0.60
Low-density cholesterol	97.4±32.7	118.1±28.2	0.08
Pro-BNP	465.8±154.3	467.0±30.6	0.020

ACE=angiotensin-converting enzyme; ARB=angiotensin receptor blocker; BNP=B-type natriuretic peptide; BUN=blood urea nitrogen; CAD=coronary artery disease; COPD=chronic obstructive pulmonary disease; CRP=C-reactive protein; HbA1C=hemoglobin A1C

reactive protein (CRP) was measured as 6.9±11.1 in the patient group and 1.3±1.4 in the control group. A statistically significant difference was found between the groups (*P*<0.01). Besides, a significant difference was found between the two groups in monocyte, hemoglobin A1C, and pro B-type natriuretic peptide values. Laboratory findings of the patient and control groups were summarized in Table 1. When the demographic characteristics of the two groups were examined, it was determined that there was a significant difference between hypertension and diabetes mellitus (*P*<0.01 and *P*=0.01, respectively). In the medical treatment they received, no significant difference was detected between the two groups, except angiotensin receptor blocker. When the complete blood count was evaluated between the two groups, all parameters except the monocyte ratio were found to be similar (0.6±0.14 *vs*. 0.4±0.1; *P*=0.012) (Table 1).

### Enumeration of Endothelial Progenitor Cells

The number of EPC in a million (1×10^6^) cell counts were counted by flow cytometry both in control and patient groups (Supplement). The numerical values of CD34+/CD309+, CD34+/CD146+, CD34+/CD146+/CD144+, CD309+, and CD34+ cells measured by gating in the control group are shown in Table 2.

**Supplement:** Cell numbers obtained by the flow cytometry analysis results in the patient and control groups.

	CD34, Immature progenitor cell numbers (CD45 dim gate)					Immature monocyte cell	
Control group	CD34	CD34+CD133	CD34+CD309	CD34+CD146	CD34+CD146+CD144	CD133	CD309
1	132	85	4	6	8	110	10
2	167	105	7	19	14	139	12
3	131	85	1	21	3	101	15
4	103	49	2	15	5	93	10
5	156	69	3	18	10	96	10
6	148	70	8	21	15	124	40
7	160	86	26	44	23	114	34
8	358	240	14	20	10	292	26
9	192	51	2	32	16	140	12
10	153	84	4	35	8	126	12
Patient group							
1	290	190	15	60	10	235	75
2	285	200	5	60	15	250	30
3	375	155	15	35	5	90	35
4	187	28	5	48	4	58	30
5	189	74	2	36	5	94	11
6	160	62	4	40	8	90	40
7	615	317	5	36	10	400	30
8	227	102	1	45	6	134	14
9	107	42	3	43	7	44	21
10	181	70	3	53	6	87	15
11	155	46	3	20	2	60	7
12	130	51	3	21	6	82	13
13	250	137	3	21	4	155	16
14	205	97	5	15	4	157	24
15	126	31	5	23	11	43	19
16	310	98	10	47	11	128	36
17	285	137	10	36	11	191	21
18	240	89	3	29	4	107	15
19	150	69	8	50	8	102	28
20	238	88	8	50	20	508	14
21	115	43	8	60	30	84	28
22	104	42	6	22	10	60	14
23	288	23	6	38	6	50	36
24	162	44	8	36	10	54	30
25	144	50	24	102	40	86	40
26	450	324	16	52	22	360	54
27	240	148	4	46	22	22	16
28	247	127	30	168	52	176	53

**Table 2:** Comparison of progenitor cell counts in one million cell counts in patients (aneurysm diameters) and control samples.

Marker	Aneurysm group			Control group	*P*-value
	All, n=30	Diameter < 48 mm, n=15	Diameter ≥ 48 mm n=15	n=10	
CD34+	216 (153.7-285.7)	187 (104-615)	240 (115-450)	154.5 (131.7-173.2)	0.058
CD309+	26 (15-35.2)	24 (7-40)	28 (13-75)	12 (10-28)	0.024
CD34+/CD146+	41.5 (28.5-52.2)	36 (15-102)	46 (21-168)	20.5 (17.2-32.7)	< 0.01
CD34+/CD146+/CD144+	9 (5-16.2)	6 (1-40)	11 (4-52)	10 (7.2-15.2)	0.656
CD146+/CD144-	30.5 (23.5-36)	-	-	9.5 (10-17.5)	< 0.01
CD34+/CD309+/CD14-	5.5 (3-11.2)	5 (2-24)	8 (1-30)	4 (2-9.5)	0.272
DHR 123 (SI)	58.3±6.8			62.4±8.5	0.154

DHR=dihydrorhodamine; SI=stimulation index

The mean EPC numbers were found to be 41.5/10^6^ and 20.5 /10^6^ in the patient and control groups, respectively, in which the number of CD34+/CD146+ EPC was significantly higher in both patient and healthy volunteers. Also, the number of CD309+ precursor monocytic cells was significantly higher in the patient group (*P*=0.024). There was no statistically significant difference between the number of cells expressing between the patient and control groups.

**Fig. 1:**
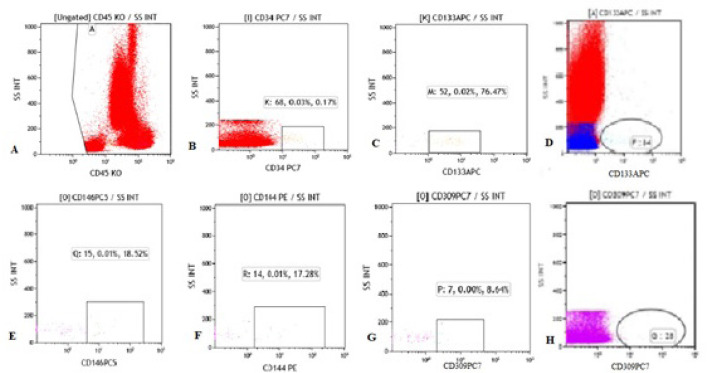
Representation of flow cytometry gating models of endothelial progenitor cell (EPC) markers at the control sample (C1). Cells were labeled with fluorescent marker antibodies targeting the hematopoietic progenitor, endothelium, leukocyte, and monocyte cell. According to expression levels of antibodies, EPCs were gated and evaluated, and the number of EPCs was calculated on both total cell count and % gated values in flow cytometry analysis.

**Fig. 2:**
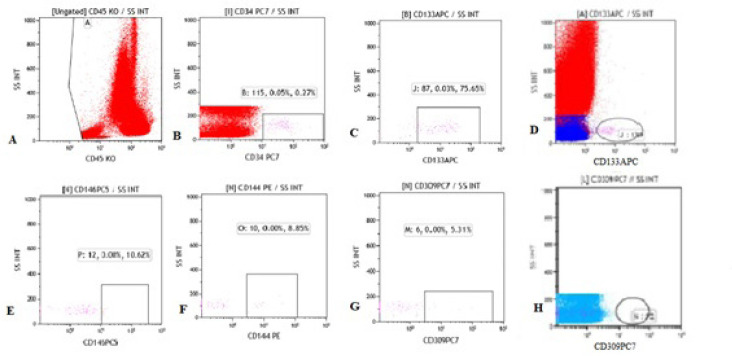
Representation of flow cytometry gating models of endothelial progenitor cell (EPC) markers at the patient sample (P1). Cells were labeled with fluorescent marker antibodies targeting the hematopoietic progenitor, endothelium, leukocyte, and monocyte cell. According to expression levels of antibodies, EPCs were gated and evaluated, and the number of EPCs was calculated on both total cell count and % gated values in flow cytometry analysis.

When the relationship between EPC numbers and clinical characteristics of the patients was evaluated, no statistically significant correlation was determined. However, there was a statistically positive correlation between aortic diameter measured by echocardiography and numbers of CD146+/CD144+ EPC. Comparing the number of EPC in patients with aneurysm diameter ≥ 48 mm with those with aneurysm diameter < 48 mm, CCD34+/CD146+/CD144+ cells were found to be significantly higher in patients with aneurysm diameter > 48 mm. For the other parameters, no significant difference was found between the two groups (Table 2). Flow cytometry results of the specific markers of EPCs according to the total cell count of the expression levels of controls and patients are shown in Figures 1 and 2.

### Neutrophil Function Test

**Fig. 3:**
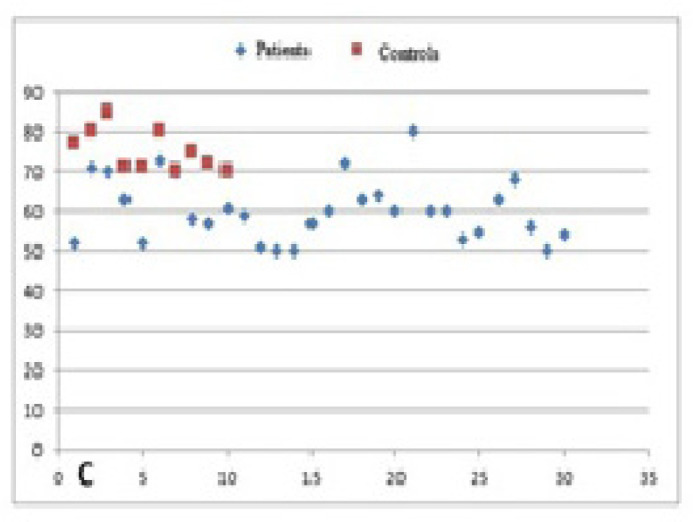
Comparison of the dihydrorhodamine 123 test stimulation index values between the control and patient groups.

**Fig. 4:**
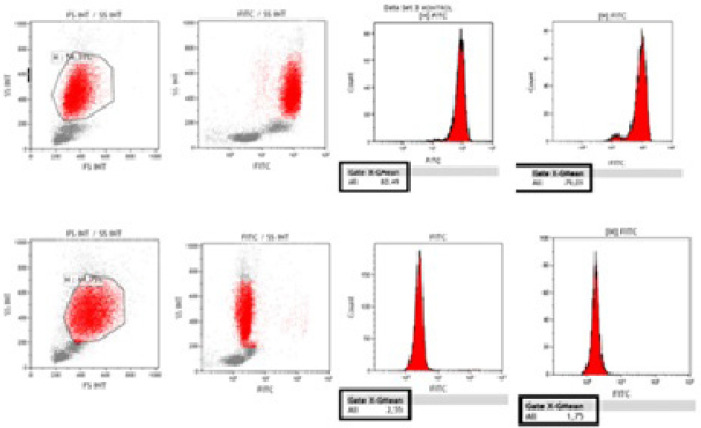
Dihydrorhodamine (DHR) histogram pattern showing patient (P1) and control (C1) results with mean stimulation index (SI) values. Phorbol 12-myristate 13-acetate (PMA) is a suitable stimulating agent for determining the function of neutrophils, nicotinamide adenine dinucleotide phosphate oxidase enzyme, and helps to determine the activation of neutrophils. DHR, on the other hand, is a fluorescent-acting antibody that indicates whether PMA activates neutrophils in the fluorescein isothiocyanate channel. Expression levels of neutrophils before stimulation with the upper panel were determined according to the increasing fluorescence intensity in the neutrophils after PMA stimulation with the lower panel. Histogram and point plots of the control sample were created by calculating the SI values of the patient and control samples exposed to the DHR 123 test PMA. By comparing both patient and control samples, P-value (patient [n=30], control [n=10]; P=0.154) was obtained. The mean SI value was found to be 60.40±7.86 in the patient group and 75.10±5.21 in the control group (P<0.01).

NADPH oxidase enzyme capacity evaluated with SI in patients was 60.4±7.8, and in the control group it was 75.1±5.2, by DHR assay (Figure 3). It was found that mean SI values were significantly lower in the patient group than in the control group (Figure 3). SI values of the DHR test in patients and controls are shown by flow cytometry analysis (Figure 4).

## DISCUSSION

The studies showed that inflammation and tissue degeneration, which are associated with many chronic diseases, have a great deal on the formation and progression of aortic aneurysms^[10^,^13^,^14]^. Inflammation of the vascular layers and destruction of extracellular matrix proteins are known to play a role in the pathogenesis of aortic aneurysm as well as hemodynamic factors^[14^,^15]^. ROS are involved in the pathogenesis of many chronic diseases such as atherosclerosis and hypertension^[15]^. ROS are molecules that can also occur during normal metabolism and cause a local inflammatory response, causing progressive cell and tissue death (oxidative stress). ROS level in the organism is regulated by adjusting the expression of antioxidant and oxidant enzymes to maintain homeostatic balance. The mechanisms of ROS production in non-phagocytic cells are puzzling, but NADPH oxidase enzyme isomers play a key role. Inspired by this situation, DHR 123 test was performed in phagocytic cells in our study, and when the SI values, which are an indirect indicator of general ROS generation capacity, in both patient and control groups were compared, it was found that SI values were significantly lower in the patient group (*P*<0.01).

DHR 123 test is the most sensitive flow cytometric indicator used to detect oxidative burst in peripheral neutrophils. This test provides information on the ROS production capacity of NADPH oxidase enzyme. SI values used for interpretation of the DHR 123 test should be between 50-100 in healthy individuals^[5^,^16]^. In our study, the mean SI value was found to be 60.40±7.86 in the patient group and 75.10±5.21 in the control group (*P*<0.01). This difference does not mean that ROS production is less in the patient group. The DHR 123 test provides information about ROS production capacity of the NADPH oxidase enzyme in an individual. These decreased capacities, which also occur in aortic aneurysm patients, may need to increase the duration of the activity of NADPH oxidase enzymes in phagocytic cells and its isomers in non-phagocytic cells, especially NOXs in vascular endothelium. Prolonged activity of NADPH oxidase enzyme may cause tissue damage and inflammation.

There are studies in the literature evaluating the relationship between oxidative stress and hypertension, smoking, male gender, dyslipidemia, and coronary artery disease (CAD), which are among the major risk factors for the development of aortic aneurysm^[17^,^18]^. Again, smoking, hyperlipidemia, and the presence of concomitant CAD have been shown to stimulate the production of superoxide in the aortic aneurysm^[19]^. In our study, no significant difference was found between the patients with TAA and with/without hypertension.

There are several studies in the literature examining the relationship between aortic aneurysm and EPCs. In these studies, they generally investigated CD34+/CD133+ cells in abdominal aortic aneurysm (AAA) and CD34+/CD309 + cells in ascending aortic aneurysm^[20^,^21]^. Our research is the first study in the literature to evaluate CD34+/CD146 + cells in TAA patients in addition to the findings of other studies. Studies conducted have shown that disease progression can be monitored by flow cytometric methods and that it can show how EPCs can play a role in the underlying pathogenesis^[20]^. Experimental studies have shown that circulating endothelial progenitor cells (or CEPC) contribute to the ongoing endothelial repair mechanism in the affected arterial region after balloon damage and replace new dysfunctional endothelial cells^[22]^. In our study, no statistically significant difference was observed in CD34+/CD309+ cells between patient and control groups (*P*=0.27).

CD146 and CD144 are some of the components of the endothelial intercellular binding site. Bardin et al.^[23]^ have shown that CD146 is localized in the junction of two endothelial cells. CD146 regulates the endothelial single cell layer, controls the paracellular permeability, and provides the connection with other neighboring cells as it is associated with actin forming the cell skeleton. There are no publications in the literature that study CD146+ and CD144+ microparticles or cells in TAA. However, when we look at the pathophysiology of aortic aneurysm, inflammation plays an important role, and endothelial cell dysfunction occurs. CD146 level was also studied in patients with a small number of chronic heart disease, and plasma CD146 level was found to be high. These cells are increasing in number to repair the area in case of any damage. CD34+/CD146+ cell count was found to be significantly higher in patients with TAA in our study (*P*<0.01). Therefore, our results are consistent with the literature. In the study conducted by Hosseinzadeh et al.^[24]^, it was reported that CD144+ endothelial microparticles were found to be higher in patients with vascular risk factors (hypertension, diabetes, hyperlipidemia, stroke, CAD, and smoking) than in those with normal cognitive function, without vascular risk factors. CD144+ cells are involved in the adhesion of homophilic cells and again in case of any damage to the endothelium, the number of these cells is increased to repair the damaged region. In our study, the number of CD34+/CD146+/CD144+ cells were significantly higher in patients with aneurysm diameter ≥ 48 mm than in those with diameter < 48 mm (*P*<0.05). This may be due to the increase in the number of CD146+/CD144+ cells involved in regeneration in the endothelium due to the increased diameter of the damaged region, and thus the increased number of precursor CD34+/CD146+/CD144+ cells in the blood.

In our study, monocytic blood cells were found to be significantly higher in the patient group. While monocytic cells were significantly higher in the whole blood count in the patient group and CD309+ cells were higher in flow cytometric analysis, it was thought that monocytic/dendritic cells could play a role in the pathogenesis of TAA. Since monocytic cells were involved in both degenerative and regenerative processes in our patient group, they were found to be significantly higher in our patients.

One of the risk factors of aortic aneurysm development is the high CRP level. Studies have shown that CRP levels in patients with symptomatic and ruptured AAA are significantly higher than in those with asymptomatic aneurysms^[25]^. In our study, CRP level was found to be significantly higher in our patient group than in the control group (*P*<0.01).

### Limitations

Our study has some limitations. First, although the control group was chosen with similar characteristics to our patient group, it was lower in number than the patient group. And secondly, this is a single-center study.

## CONCLUSION

Our results showed that the number of EPCs is significantly higher in aortic aneurysm patients and may have a role in disease progression. The crosstalk between NADPH oxidase activity and EPC number may be useful as a parameter to explain the clinical progression of TAA. Our research is the first study in the literature to evaluate CD34+/CD146+ cells in TAA patients The presence of CD34+/CD146+ cells, which have important functions in the binding region between endothelial cells, and the close association of these cells with infiltration, as demonstrated in other studies, showed that CD34+/CD146+ cells may be an important marker in this disease. Our study is a guiding light for future studies with larger numbers of patients. However, there are many unknown aspects of the pathogenesis of TAA. To diagnose this disease with serious complications early and to prevent its progression, these pathways should be clarified. These decreased capacities of NADPH oxidase enzymes activity in TAA may be compensated by increasing the duration of the activity of it in phagocytic cells and its isomers in non-phagocytic cells, especially NOXs in vascular endothelium. So, prolonged activity of NADPH oxidase enzyme with continuous ROS secretions may cause tissue damage and inflammation.ww

**Table:** 

Authors' roles & responsibilities	
DE	Substantial contributions to the conception or design of the work; or the acquisition, analysis, or interpretation of data for the work; drafting the work or revising it critically for important intellectual content; final approval of the version to be published
BB	Substantial contributions to the conception or design of the work; or the acquisition, analysis, or interpretation of data for the work; drafting the work or revising it critically for important intellectual content; final approval of the version to be published
SK	Substantial contributions to the conception or design of the work; or the acquisition, analysis, or interpretation of data for the work; drafting the work or revising it critically for important intellectual content; final approval of the version to be published
SÖ	Substantial contributions to the conception or design of the work; or the acquisition, analysis, or interpretation of data for the work; drafting the work or revising it critically for important intellectual content; final approval of the version to be published
AT	Substantial contributions to the conception or design of the work; or the acquisition, analysis, or interpretation of data for the work; drafting the work or revising it critically for important intellectual content; final approval of the version to be published
ZC	Substantial contributions to the conception or design of the work; or the acquisition, analysis, or interpretation of data for the work; drafting the work or revising it critically for important intellectual content; final approval of the version to be published
JS	Substantial contributions to the conception or design of the work; or the acquisition, analysis, or interpretation of data for the work; drafting the work or revising it critically for important intellectual content; final approval of the version to be published
MTI	Substantial contributions to the conception or design of the work; or the acquisition, analysis, or interpretation of data for the work; drafting the work or revising it critically for important intellectual content; final approval of the version to be published
NK	Substantial contributions to the conception or design of the work; or the acquisition, analysis, or interpretation of data for the work; drafting the work or revising it critically for important intellectual content; final approval of the version to be published
MYK	Substantial contributions to the conception or design of the work; or the acquisition, analysis, or interpretation of data for the work; drafting the work or revising it critically for important intellectual content; final approval of the version to be published

## References

[1] Aggarwal S, Qamar A, Sharma V, Sharma A (2011). Abdominal aortic aneurysm: A comprehensive review. Exp Clin Cardiol.

[2] Steckmeier B (2001). Epidemiology of aortic disease: aneurysm, dissection,
occlusion. Radiologe.

[3] Wiernicki I, Parafiniuk M, Kolasa-Wołosiuk A, Gutowska I, Kazimierczak A, Clark J, Baranowska-Bosiacka I, Szumilowicz P, Gutowski P (2019). Relationship between aortic wall oxidative stress/proteolytic
enzyme expression and intraluminal thrombus thickness indicates a novel
pathomechanism in the progression of human abdominal aortic
aneurysm. FASEB J.

[4] Roos D, van Bruggen R, Meischl C (2003). Oxidative killing of microbes by neutrophils. Microbes and infection.

[5] Köker MY, Camcıoğlu Y, van Leeuwen K, Kılıç SŞ, Barlan I, Yılmaz M (2013). Clinical, functional, and genetic characterization of chronic
granulomatous disease in 89 Turkish patients. J Allergy Clin Immunol.

[6] Dikalov SI, Dikalova AE, Bikineyeva AT (2008). Distinct roles of Nox1 and Nox4 in basal and angiotensin
II-stimulated superoxide and hydrogen peroxide production. Free radical biology & medicine.

[7] Malecki C, Hambly BD, Jeremy RW, Robertson EN (2020). The Role of Inflammation and Myeloperoxidase-Related Oxidative
Stress in the Pathogenesis of Genetically Triggered Thoracic Aortic
Aneurysms. Int J Mol Sci.

[8] Friedrich EB, Walenta K, Scharlau J, Nickenig G, Werner N (2006). CD34-/CD133+/VEGFR-2+ endothelial progenitor cell subpopulation
with potent vasoregenerative capacities. Circ Res.

[9] Davies MJ (1998). Aortic aneurysm formation: lessons from human studies and
experimental models. Circulation.

[10] Spartalis E, Spartalis M, Athanasiou A, Paschou SA, Patelis N, Voudris V, Iliopoulos DC (2020). Endothelium in Aortic Aneurysm Disease: New
Insights. Curr Med Chem.

[11] George Jacob, Shmilovich Haim, Deutsch Varda, Miller Hylton, Keren Gad, Roth Arie (2006). Comparative Analysis of Methods for Assessment of Circulating
Endothelial Progenitor Cells.

[12] Köker MY, Sanal O, de Boer M, Tezcan I, Metin A, Tan C (2006). Skewing of X-chromosome inactivation in three generations of
carriers with X-linked chronic granulomatous disease within one
family. Eur J Clin Invest.

[13] Shimizu K, Mitchell RN, Libby P (2006). Inflammation and cellular immune responses in abdominal aortic
aneurysms. Arteriosclerosis, thrombosis, and vascular biology.

[14] Brophy CM, Reilly JM, Smith GW (1991). The role of inflammation in nonspecific abdominal aortic aneurysm
disease. Annals of vascular surgery.

[15] Busch A, Chernogubova E, Jin H, Meurer F, Eckstein HH, Kim M, Maegdefessel L (2018). Four Surgical Modifications to the Classic Elastase Perfusion
Aneurysm Model Enable Haemodynamic Alterations and Extended Elastase
Perfusion. Eur J Vasc Endovasc Surg.

[16] Xiong W, Mactaggart J, Knispel R (2009). Inhibition of reactive oxygen species attenuates aneurysm
formation in a murine model. Atherosclerosis.

[17] Griese DP, Ehsan A, Melo LG (2003). Isolation and transplantation of autologous circulating
endothelial cells into denuded vessels and prosthetic grafts: implications
for cell-based vascular therapy. Circulation.

[18] Newman KM, Jean-Claude J, Li H (1994). Cytokines that activate proteolysis are increased in abdominal
aortic aneurysms. Circulation.

[19] Guzik B, Sagan A, Ludew D (2013). Mechanisms of oxidative stress in human aortic
aneurysms—association with clinical risk factors for atherosclerosis and
disease severity. International journal of cardiology.

[20] Parietti E, Pallandre J-R, Deschaseaux F (2011). Presence of circulating endothelial progenitor cells and levels
of stromal-derived factor-1α are associated with ascending aorta
aneurysm size. European Journal of CardioThoracic Surgery.

[21] Sho E, Sho M, Nanjo H (2004). Hemodynamic regulation of CD34+ cell localization and
differentiation in experimental aneurysms. Arteriosclerosis, thrombosis, and vascular biology.

[22] Walter DH, Rittig K, Bahlmann FH (2002). Statin therapy accelerates reendothelialization: a novel effect
involving mobilization and incorporation of bone marrow-derived endothelial
progenitor cells. Circulation.

[23] Bardin N, Anfosso F, Massé J-M (2001). Identification of CD146 as a component of the endothelial
junction involved in the control of cell-cell cohesion. Blood.

[24] Hosseinzadeh S, Noroozian M, Mortaz E (2018). Plasma microparticles in Alzheimer’s disease: The role of
vascular dysfunction. Metabolic brain disease.

[25] De Haro J, Acin F, Bleda S, Varela C, Medina FJ, Esparza L (2012). Prediction of asymptomatic abdominal aortic aneurysm expansion by
means of rate of variation of C-reactive protein plasma
levels. J Vasc Surg.

